# Hatching enzymes disrupt aberrant gonadal degeneration by the autophagy/apoptosis cell fate decision

**DOI:** 10.1038/s41598-017-03314-7

**Published:** 2017-06-09

**Authors:** Tapas Chakraborty, Sipra Mohapatra, Megumi Tobayama, Kayoko Ohta, Yong-Woon Ryu, Yukinori Kazeto, Kohei Ohta, Linyan Zhou, Yoshitaka Nagahama, Takahiro Matsubara

**Affiliations:** 1 South Ehime Fisheries Research Center, Ehime University, Ainan, 798-4206 Japan; 2 National Research Institute of Aquaculture, Oita, 879-2602 Japan; 3 Laboratory of Marine Biology, Kyushu University, Fukouka, 812-8581 Japan; 4 Key Laboratory of Freshwater Fish Reproduction and Development, Southwest University, Chongqing, 400715 China; 5 Institution for Collaborative Relations, Ehime University, Matsuyama, 790-8577 Japan

## Abstract

Environmental stressors, gonadal degenerative diseases and tumour development can significantly alter the oocyte physiology, and species fertility and fitness. To expand the molecular understanding about oocyte degradation, we isolated several spliced variants of Japanese anchovy hatching enzymes (AcHEs; ovastacin homologue) 1 and 2, and analysed their potential in oocyte sustenance. Particularly, *AcHE1b*, an ovary-specific, steroid-regulated, methylation-dependent, stress-responsive isoform, was neofunctionalized to regulate autophagic oocyte degeneration. *AcHE1a* and *2* triggered apoptotic degeneration in vitellogenic and mature oocytes, respectively. Progesterone, starvation, and high temperature elevated the total degenerating oocyte population and *AcHE1b* transcription by hyper-demethylation. Overexpression, knockdown and intracellular zinc ion chelation study confirmed the functional significance of *AcHE1b* in autophagy induction, possibly to mitigate the stress effects in fish, via ion-homeostasis. Our finding chronicles the importance of *AcHE*s in stress-influenced apoptosis/autophagy cell fate decision and may prove significant in reproductive failure assessments, gonadal health maintenance and ovarian degenerative disease therapy.

## Introduction

Sustainable species maintenance largely depends on the quality of eggs or oocytes. Gonadal atresia and oocyte degeneration are steroid responsive energy-sustaining phenomena, and are also widely acknowledged egg quality and reproductive success indicators in vertebrates^1, 2^. High throughput sequencing and functional genomics studies using zebrafish, salmonids and flat fish, emphasizes the importance of follicular atresia in vertebrate reproduction^3–5^. Despite the importance of germ cell/oocyte in reproduction and ovarian atresia^3–5^, little else is known about the molecular paradigm of atresia in oocytes.

Programmed cell death (PCD), first coined by Lockshin and Williams in 1964, is functionally important for several physiological processes, including gonadal maintenance and atresia^6^. Two pathways of PCD, apoptosis and autophagy, have been well characterized in several species^7^. While apoptosis and autophagy may occur together or distinctly, the expected outcome is the physiological removal of marked cells, leading to the development of new structures (morphogenesis), removal of damaged cells, and tissue homeostasis.

In vertebrate ovaries, gonadotropins, oestrogens, growth hormones, growth factors (IGF, EGF/TGFα, basic FGF), cytokines (interleukin-1β) and nitric oxide act in concert to ensure the survival of gonia and pre-ovulatory follicles^6^. However, in contrast, the ovarian androgens, interleukin-6 and gonadal GnRH-like peptides act as apoptotic factors^8^. Moreover, intra- and extracellular ion homeostasis of several divalent cations (Zn, Ca, etc.) have been reported to alter cellular atresia^8, 9^. Several metalloproteinases/choriolytic genes (ovastacin, hatching enzyme (HE), etc.) are also known to induce atresia in vertebrates^10^. In human, SAS1B (a.k.a ovastacin) is expressed in growing oocytes and uterine tumors, and has been postulated to be a major candidate for uterine tumor therapy^11, 12^. However, despite speculation and ovarian expression, the involvement of choriolytic genes in oocyte degeneration is still largely unknown^11–13^. Indepth knowledge of such phenomenon will advance the modus operandi required for egg quality assessment, gonadal degenerative disease therapy, and targeted ovarian or uterine cancer/tumor treatment.

Genomic duplication and alternative splicing are the two major driving forces for evolution^14, 15^. Evolutionarily, although fish occupies the lowest pane of vertebrate clade, it also possesses several attributes necessary to address complex research questions, thereby making these aquatic models efficacious for human disease study. Fish ovaries are a suitable experimental model system for studying PCD, due to the presence of both atretic and non-atretic oocytes^5^. Surprisingly, both human and rodent ovastacins are more similar to teleostean HE, than Xenopus HE^10^, thus rendering fish as an excellent alternative for exploring the pros and cons of mammalian ovarian atresia. Therefore, the present investigation was conducted to gain insight into the nature of the underlying molecular relationship of oocyte degeneration and hatching enzymes. The conserved oocyte specific expression profiles of hatching enzyme homologues across vertebrates urged us to focus only on the oocytes and not on the follicles.

Japanese anchovy (*Engraulis japonicus*) is a fast growing, heterogametic, primitive marine daily-breeding teleost, and possesses excellent demarcation between atretic and non-atretic oocytes. This fish has relatively large sized gonads, which gives it a unique advantage over the other relatively established models, i.e. zebrafish and medaka, to facilitate multiple experiments from the same gonad without any individual variations. Moreover, the previously identified five AcHE homologues were found to have significant role in embryonic chorion lysis^16^, which led us to hypothesize that at least some of the homologues might be essential for gonadal atresia, especially oocyte degeneration.

## Results

### Epigenetically regulated alternative splicing of HEs

**Figure 1:**
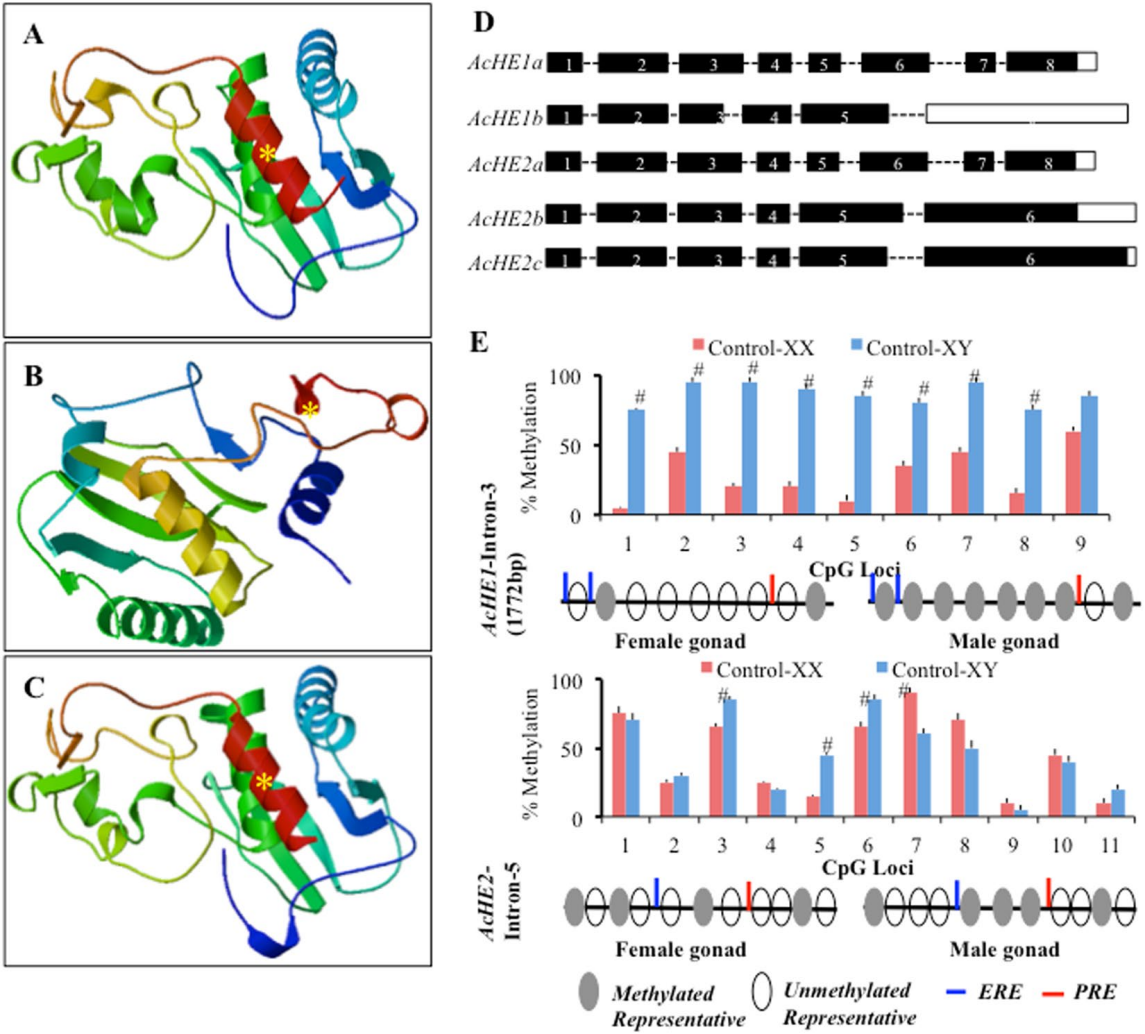
Alternative splicing of *AcHE*s. *In silico* structural analysis of putative proteins depicted significant structural differences among AcHE1a (**A**), 1b (**B**) and 2a (**C**), especially in protein folding pattern in C-terminal region. Note: yellow asterisk (*) indicates the astacin (red ribbon shaped) domain. Schematic diagram show the alternative splicing of AcHE1 and 2, where, black boxes represents exon and dotted line indicates intron (**D**). Strong male dominated methylation of *AcHE1*, and not *AcHE*2, (**E**) was observed in Japanese anchovy adult gonads. Note: The graphical data are presented as percent mean ± SE; asterisk ‘#’ denotes significant sexual differences at each loci (P < 0.01); N = 15 individuals per sex, randomly collected from three distinct mature fish population @ 5/population; and the schematic diagram depicting the general methylation profile in female/male gonad and putative ERE (oestrogen responsive element) and PRE (progesterone responsive element) sites.

Gonadal atresia, particularly chorionic degradation, involves a complex enzymatic regulation, similar to embryonic hatching^16, 17^. Donato *et al*.^13^ suggested the existence of hatching enzymes in atretic ovaries; however, the mechanisms underlying the hatching enzymes regulated gonadal degeneration has not yet been elucidated in detail. Furthermore, the existence of multiple isoforms of teleostean HEs makes it difficult to establish their specific importance in oocyte degeneration^17, 18^. In an attempt to gain additional insight, we measured the gonadal expression profiles of all existing *AcHE*s (Supplementary Fig. 1), and selected *AcHE1* and *2* for further analysis. Alternative splicing is a useful mechanism for the post-transcriptional functional diversification of any specific gene, and plays a critical role in sexual development, ovarian maintenance and cell death, in various organisms^15, 19–23^. Thus, we explored the ovarian full-length cDNA library with various *AcHE*-specific primers and isolated spliced variants of *AcHE1* and *2* (hereafter designated as *AcHE1a*, *1b*, *2a*, *2b* and *2c*, consisting of 273 (30.99KD), 208 (23.58KD), 276 (31.16KD), 300 (34.05KD) and 293aa (35.21KD), respectively) that possessed 3′ UTR of different sizes. Although all the isolated *AcHE* isoforms clustered into the type 1-hatching enzyme group (Supplementary Fig. 1), only *AcHE1a* and *2c* were identical with the pre-reported *AcHE1* and *2*, respectively^16^ (Supplementary Table 1). Alternatively spliced variants of high coriolytic enzyme (HCE) were also recorded in medaka, which were clustered into the same phylogenetic group (Supplementary Fig. 1), and showed predominant ovarian expression (Data not shown). Our *in silico* putative protein analysis also showed different structural and folding patterns amongst the alternatively spliced isoforms (Fig. 1A–C, Supplementary Table 2). Differences in C-terminal lengths and alterations in the Astacin-extended-Zinc-binding domain (HEXXHXXGXXHEXXRXDR)^16^ suggests that varying translational differences, i.e. protein localization, protein folding, etc., are responsible for the functional diversity^15^.

In order to clarify the role of the alternatively spliced isoforms in anchovy development and maturation, we evaluated tissue-biased mRNA profiles during adulthood. *AcHE1a*, *2a*, *2b* and *2c* were predominant in the ovary and testis, whereas *AcHE1b* showed ovary-specific expression (Supplementary Fig. 2). Sex-dependent genomic methylation has been reported to regulate alternative splicing in various organisms^24–27^. To investigate further, we analyzed several intronic loci (depicted *in silico*) near the alternative splicing sites of both *AcHE1* and *2* genes (Fig. 1D,E). More variable methylation was observed in *AcHE1*-intron-3 and *AcHE2*-intron-5 (harbouring alternative splicing sites) than their respective preceding introns (Fig. 1E, Supplementary Fig. 2). However, only *AcHE1*-intron-3 consistently depicted a sexually dimorphic methylation pattern (Fig. 1E), which proves that female-dominated *AcHE1b* expression is an outcome of the sex-biased methylation^24–27^.

**Figure 2:**
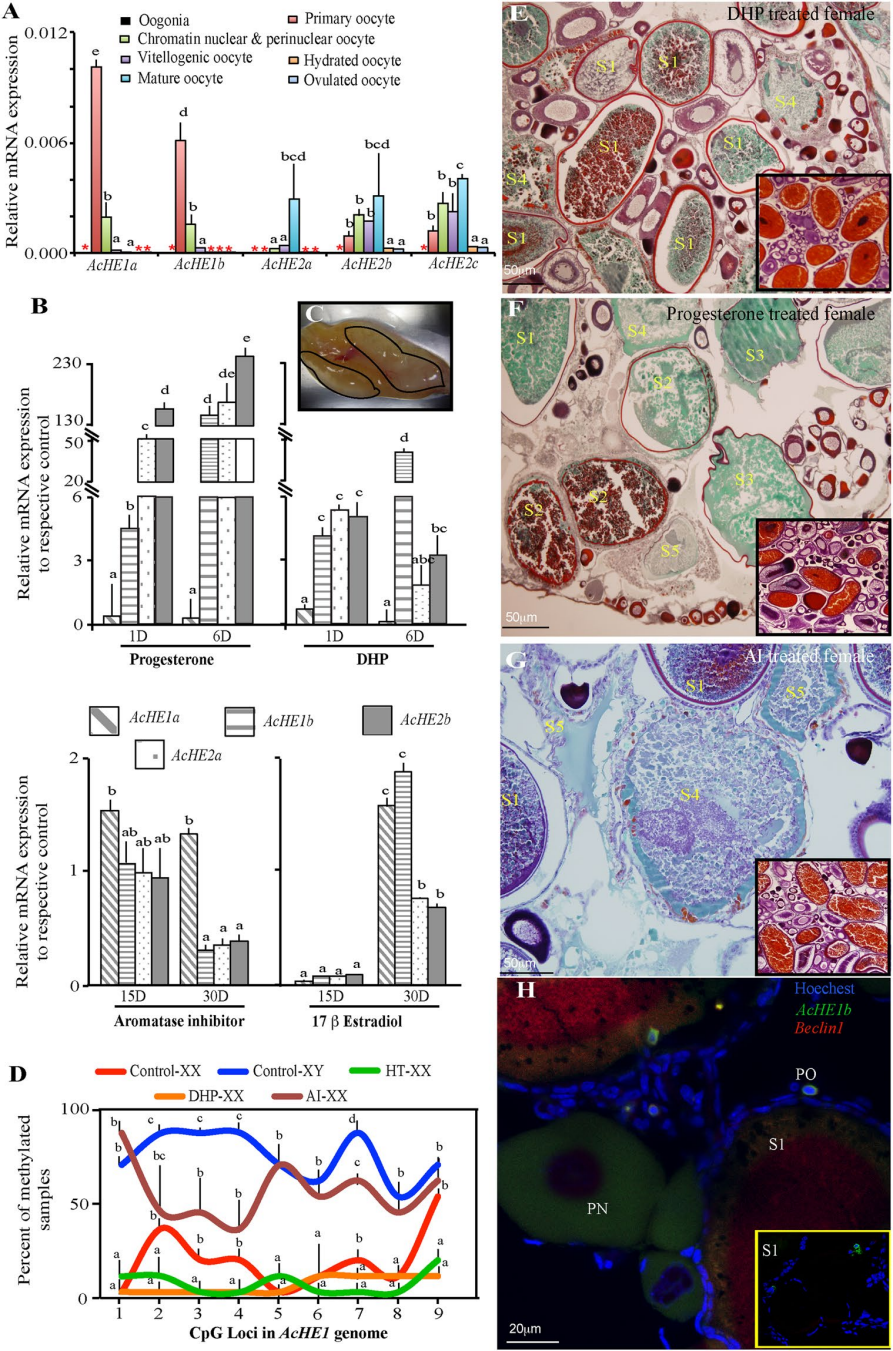
Steroidogenic alterations of *AcHE* in ovary. Quantitative PCR (qPCR) profiling determined the oocyte-stage responsive expression of *AcHE* isoforms in adult gonad (**A**). Progesterone treated gonads exhibited higher amplitude of *AcHE1b*, *2a* and *2b* transcription (**B**), and overall ovarian degeneration (**C**, black boundary) than other treatments (N = 12). The graphs are drawn using the ratio between treatment and controls (each separately normalized with respective internal control) of same time and stage. Sex steroid modulation altered the epimethylation status (N = 15) of *AcHE1*-intron-3 (**D**) and caused various extent of steroid responsive oocyte degeneration (**E**–**G**), thus suggesting that ovarian maturation and *AcHE* productions are interrelated. *Fluorescent ISH* (*FISH*) displayed the co-localization of *AcHE1b* and *Beclin1* in various stages of oocytes of DHP treated gonad, (**H**). Note: Data are presented as mean ± SEM, and different letters (a, b, etc.) denotes significant differences at P < 0.05; significance was separately calculated for each loci in graph D, while for other graphs, the significance was calculated for each group. S1–S5 represents different atretic stages (Supplementary Fig. 3); PO- Primary oocyte; PN- Peri-nuclear oocyte; Inset microphotographs represents the respective control.

The stage-specific occurrence of different spliced isoforms was observed during the course of ovarian development, with *AcHE1a* and *1b* being abundant in immature ovaries, *AcHE2a* in atretic ovaries, and *AcHE2b* and *2c* in all gonadal stages (Supplementary Fig. 2). A more comprehensive analysis using isolated oocytes (Fig. 2A) and *in situ hybridization* (*ISH*) (Supplementary Fig. 2) demonstrated that *AcHE1* isoforms were predominant in immature and pre-atretic oocytes, while *AcHE2* isoforms were mainly restricted to atretic oocytes. *AcHE1* and *2* isoforms, but not *AcHE3*, *4* and *5*, were undetected in the hydrated or ovulated oocytes (Supplementary Fig. 1). The varying expression patterns of *AcHEs* suggest that *AcHE1* and *2* variants play some role in oocyte maintenance, while the other three isoforms are involved in the hatching mechanism. Genomic duplication and alternative splicing have been identified as the main navigators of neofunctionalization in eukaryotes^14, 15, 28^. Previously, predicted cAMP- and cGMP-dependent protein kinase phosphorylation sites (important for calcium ion channel maintenance in ovaries^29^) were markedly less prevalent in *AcHE1b* (Supplementary Table 2), which, in addition to sex-biased methylation and ovary-specific expression, insinuates the differential role of *AcHE1b* in ovarian maintenance^28^. Since we observed similar expression patterns for *AcHE2b* and *2c* in different groups of female germ cells (Fig. 2A), we hereafter focused on the *AcHE1a*, *1b*, *2a* and *2b* variants and their roles in oocyte maintenance and degeneration.

### Steroid responsive modulation of HEs

In various animals, gonadal atresia is initiated just after the progesterone surge, thus highlighting progesterone-atresia inter-connections^30, 31^. The occurrence of *AcHE*s in pre-atretic and atretic ovaries raises the possibility of progesterone involvement in *AcHE* transcription. The introns of *AcHE1* and *2* were found to possess several putative half-ERE and PRE^32^ sites (Fig. 1E). Upon maturation, teleostean females undergo major hormonal changes and the presence of putative steroid recognition sites increases the possibility of potential crosstalk between *AcHE*s and sex steroids. The involvement of oestrogen and progesterone in genomic demethylation has also been recently reported^33^. Depending on the concentration of these steroids, the methylation pattern may change, and induce atresia. To verify our hypothesis, we performed *in vivo* progesterone and 17α-20β-dihydroxy-4-pregnen-3-one (DHP, maturation-inducing hormone in fish^34^) treatments, and detected excessive genomic demethylation and abundant transcription of *AcHE1b*, *2a*, and *2b*, along with accelerated gonadal atresia (Fig. 2B–H), while mild increase was recorded at each time points in the control counterparts (Supplementary Table 3). Contrary to our expectations, the addition/suppression of oestrogen had lesser impact on *AcHE* transcription compared to progesterone and DHP. Thus, we concluded that although oestrogen initiated differential *AcHE* transcription, progesterone is necessary for excessive *AcHE* production and possibly for further induction of atresia.

### Atresia and *AcHE* regulation

**Figure 3:**
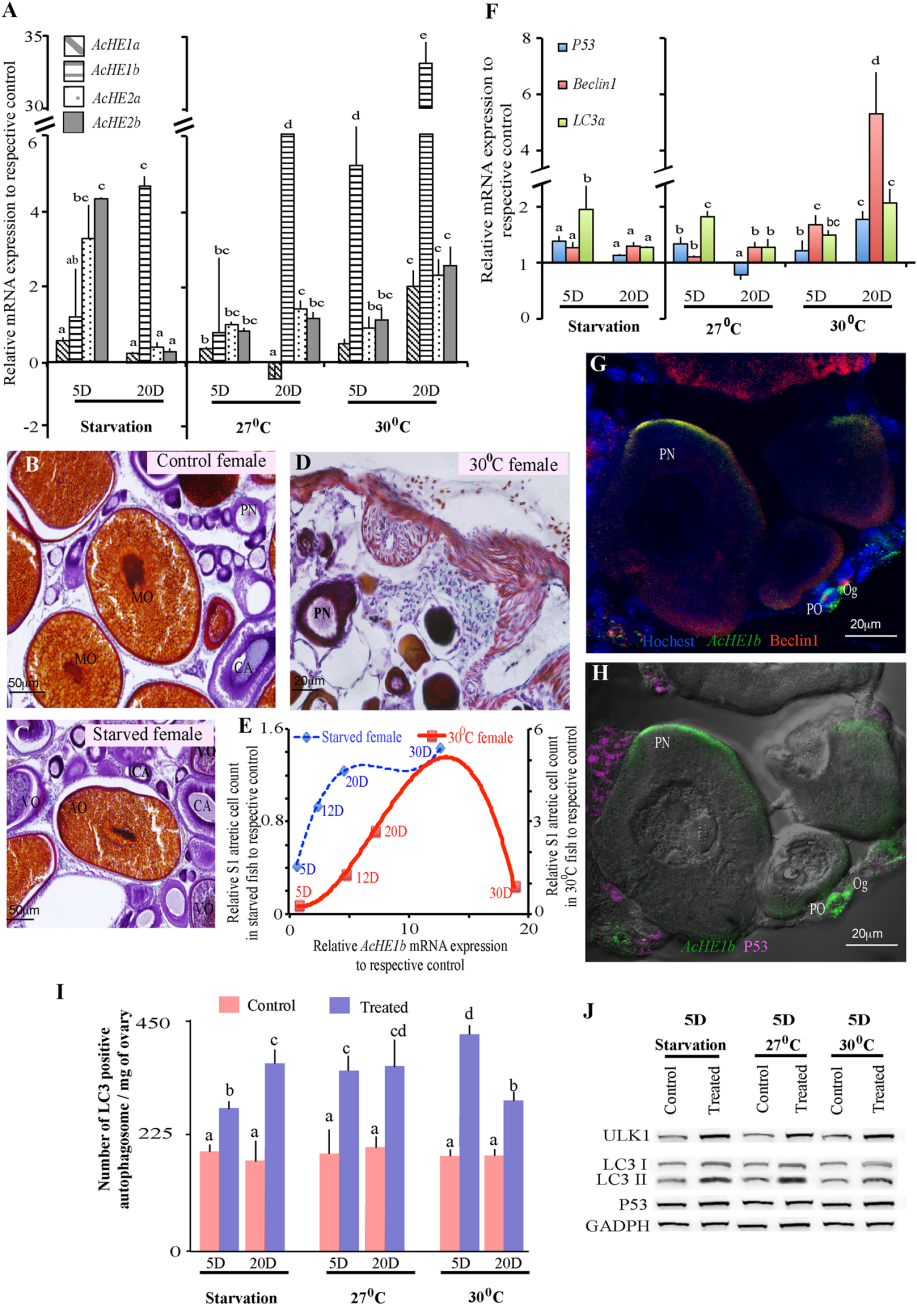
Role of *AcHE*s in ovarian atresia. Starvation and high temperature induced time-dependent *AcHE*s production (**A**), and moderate (**C**) to severe (**D**) oocyte degeneration compared to control (**B**). S1 atretic cell counts of starved (Y-axis 1) and 30 °C-reared (Y-axis 2) fish plotted against relative *AcHE1b* mRNA expression (in a polynomial trendline fit), showed positive correlation (**E**). Although, both apoptotic (*P53*) and autophagy (*Beclin1* and *LC3a*) marker genes displayed strong modulations in various treatment groups (**F**), Tricolour *FISH-*FIHC revealed that *AcHE1b* (green colour; *FISH*) co-localized only with Beclin1 (red colour, FIHC) (**G**) but not with P53 (pink colour, FIHC) (**H**), highlighting the *AcHE1b* and autophagy interaction. Both autophagosome abundance (**I**) and autophagic protein production (J), but not P53 production (**J**), further corroborates the AcHE1b specific autophagy regulation. Note: Graphical data are presented as mean ± SEM, and different letters (a, b, etc.) denotes significant differences at P < 0.05. Oogonia (Og) and Primary oocyte (PO), Peri-nuclear oocyte (PN), cortical alveoli (CA), vitellogenic (VO), mature (MO) and atretic (AO) oocytes are marked accordingly. GADPH was used as internal control for western blotting.

Gonadal atresia, a nutrient-circulating process, is activated in a synchronized manner and involves both autophagy and apoptosis^7, 35^. Starvation and high temperature (HT) are known to induce atresia in various animals^36^. In order to confirm the involvement of *AcHE*s in the initiation of autophagy/apoptosis, we examined the starved and HT-reared (27 and 30 °C) females, and observed significant time-dependent enhancements in *AcHE1b* transcription (Fig. 3A). This result further accentuated the crucial role of *AcHE1b* in the regulation of ovarian degeneration. Thereafter, we quantified the atretic cell population (Supplementary Fig. 3) and transcription of major atretic genes (*P53*, *Beclin1* and *LC3a*, Fig. 3F), in order to validate the extent of the progression of atresia. Histologically, starved and HT-reared fish showed varying levels of atresia (Fig. 3B–D), depending on the level of stress^5^. *AcHE1b* expression was strongly correlated with stage 1 (S1) atretic oocytes (a slight break in the oocyte wall and excessive cell proliferation in the surrounding granulosa (Fig. 3E, Supplementary Fig. 3, Supplementary Table 4)) and autophagy markers (*Beclin1* and *LC3a*) (Supplementary Table 5), while the other *AcHE* variants were more closely involved in apoptosis (*P53*) (Supplementary Table 5). This was further supported by the strong demethylation of *AcHE1*-intron-3 in HT-reared fish (Fig. 2B). Taken together, these results indicate that stressors induce varying levels of changes by the following pathways: (i) increased production of heat shock proteins (HSP) and their receptors, which subsequently promotes the production of several HSP-related cell death factors^37, 38^, and/or (ii) an accelerated demand for energy, which leads to glycogen breakdown, localized increments of glyceraldehyde-3-phosphate, phosphoenolpyruvate, and glucose-6-phosphate (enzymes for glucose production), and further increase in reactive oxygen species^39^. The latter pathway is known to involve autophagy instead of apoptosis^39^. We measured the amount of autophagosome formation (Fig. 3I) and autophagic protein (LC3 and ULK1) production (Fig. 3J), and found that both dramatically increased upon stress. Since we also observed stress-responsive co-induction of *AcHE1b*, *Beclin1* and *LC3a* in the degenerating ovaries/oocytes (Fig. 3F–H), we speculate that *AcHE1b* favours autophagy during atresia.

### Neofunctionalization of *AcHE1b* in autophagy

**Figure 4:**
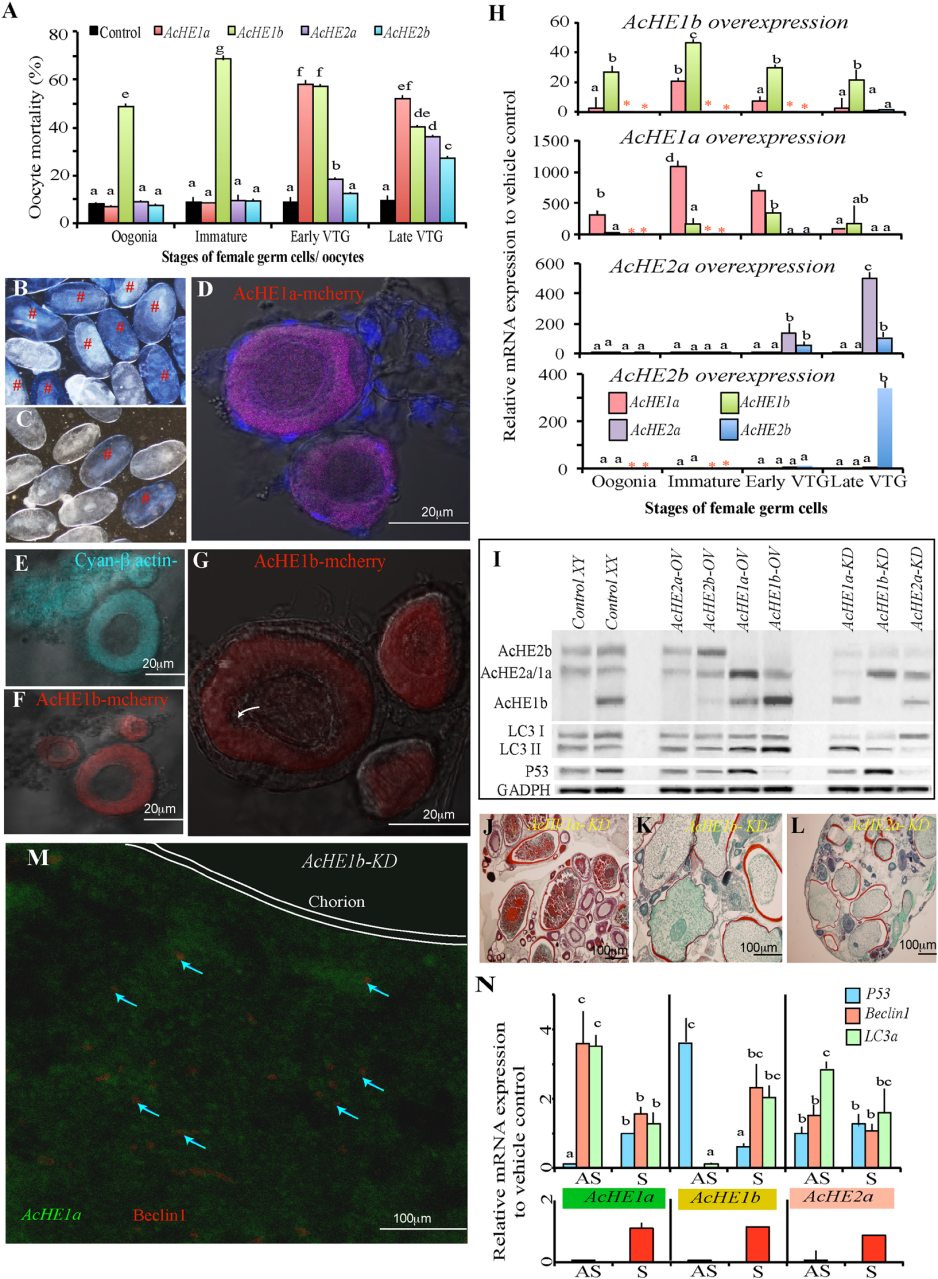
Functional analysis of *AcHE*s. *Ex vivo AcHE* OV induced various levels of oocyte mortality, highest in *AcHE1b* OV oocytes (**A**). Toluidine blue staining confirmed extensive degradation (‘#’) in *AcHE1b* OV oocytes (**B**) than their control counterpart (**C**). Live imaging of *AcHE1a* and *1b* OV immature oocytes depicted strong reporter gene expression in ooplasm (**D**,**F**) (at 24 hpt) and extensive degeneration (white arrow, at 120 hpt) in *AcHE1b* OV transfected oocytes (**G**) only. The same oocytes were examined for *cyan*-*βactin*-UTR mRNA localization to confirm the extent of transfection (**E**). QPCR analysis of different overexpressed oocytes showed that *AcHE1a*/*2a* OV could, respectively, induce *AcHE1b*/*2b* transcription and vice-versa (**H**). The specificity of overexpression and knockdown were confirmed by western blotting with eel hatching enzyme antibody (**I**). The same blots were re-analysed with LC3 and P53 antibody to respectively confirm the autophagic and apoptotic responses (**I**). GADPH was used as internal control. *In vivo AcHE1a*/*2a* KD caused significant upregulation of autophagy genes while *AcHE1b* KD induced apoptosis, confirmed by Trichrome staining (**J**–**L**), *FISH* (**M**) and qPCR (**N**), Note: Graphical data are presented as mean ± SEM, and different letters (a, b, etc.) denotes significant differences at P < 0.05. Immature oocyte contains both peri-nuclear and chromatin nuclear oocytes. VTG = vitellogenic; Blue arrow denotes Beclin1 expression.

The involvement of *AcHE1b* in oocyte maintenance and autophagy regulation was ascertained by overexpressing *AcHE1a*/*b*-*mCherry* and *AcHE2a*/*b-cyan* at different oocyte stages, and monitoring the cellular degradation for 120h. Although *AcHE1b* induced extensive damage at all oocyte stages, *AcHE1a* triggered cell death in the vitellogenic and mature oocytes (Fig. 4A–G), while *AcHE2a* and *2b* OV (overexpression) only degraded the late vitellogenic oocytes (Fig. 4A). A detailed time-dependent quantitative PCR (qPCR) analysis (24, 72 and 120h post transfection, hpt) showed that *AcHE1b* induced *Beclin1* and *LC3a* production, while all the other OV groups had an abundance of *P53* expression (Supplementary Fig. 4). *AcHE1a* and *2a* OV specifically triggered the expression of the other alternatively spliced isoforms and vice-versa (Fig. 4H–I). This might be due to some inbuilt defence mechanism against the extensive unidirectional progression of gonadal atresia. To further confirm the involvement of *AcHE1b* in autophagy, the knockdown (KD) of different *AcHE*s was performed, and the transcriptional and protein profiles of Beclin1, LC3 and P53, and ACHE production were assessed. *AcHE1a* KD gonads depicted elevated levels of *AcHE1b*, *Beclin1* and *LC3a*, while *AcHE1b* KD samples portrayed higher levels of *AcHE1a* and *P53* transcripts (Fig. 4J–N). Our fluorescent immunohistochemistry (FIHC) and western blotting data also displayed similar trend (Fig. 4M,I).

**Figure 5:**
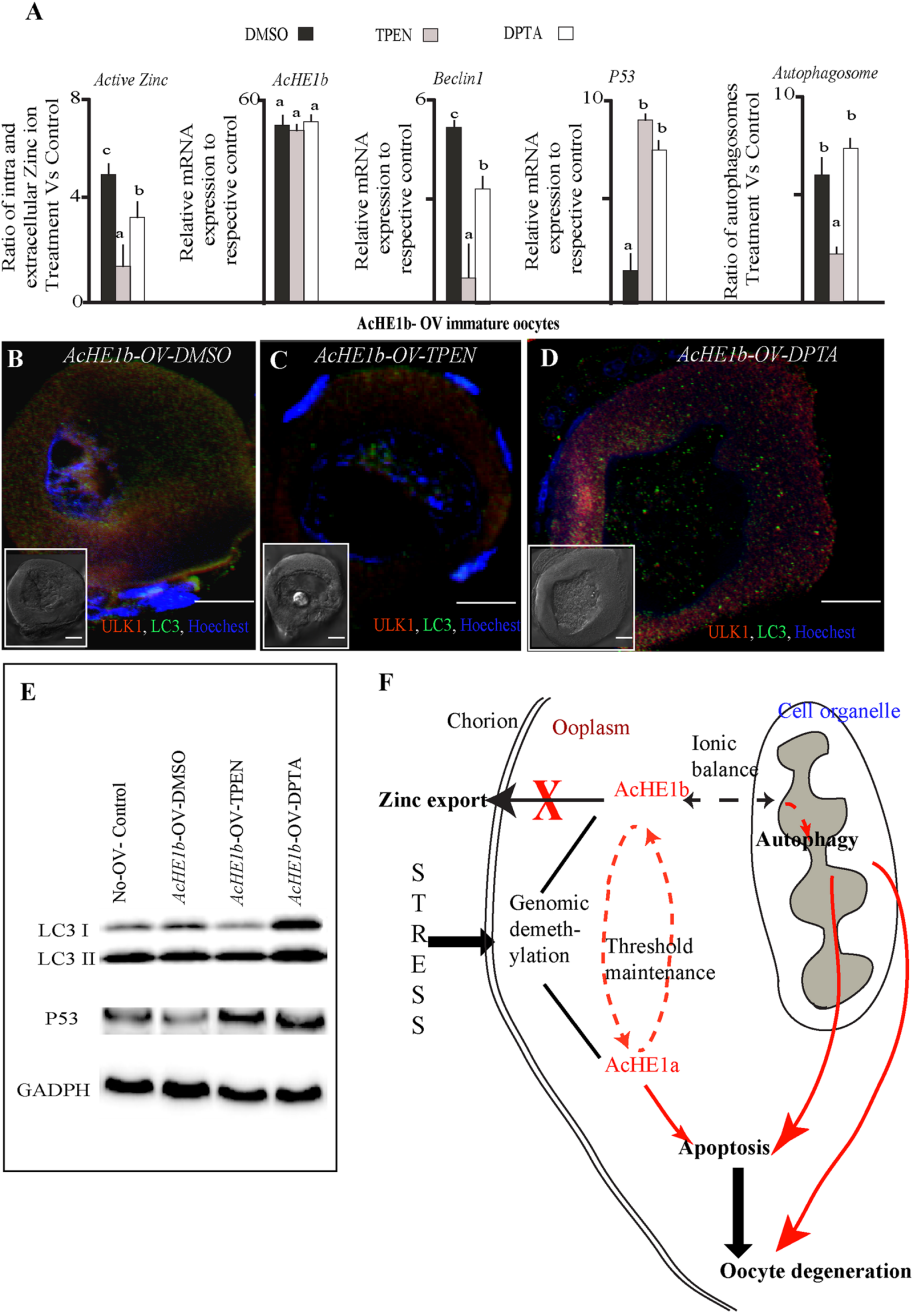
*AcHE1b* and Zinc (Zn^+2^) transport. *AcHE1b* OV immature oocytes were treated with intra (TPEN) or extra (DPTA) cellular Zn^+2^ chelator and the ratio of intra and extra cellular Zn^+2^ ion concentration, and *AcHE1b*, *Beclin1* and *P53* transcription (**A**) was measured. ULK1 and LC3 antibodies were used to confirm to extent of autophagy in DMSO (**B**), TPEN (**C**) and DPTA (**D**) treated *AcHE1b* OV oocytes. Western blotting analysis also substantiated the autophagic/apoptotic alterations (**E**). Our data suggests that *AcHE1b* regulates the Zn^+2^ ion balance, which further decides the cell fate. General mechanism of *AcHE*-atresia regulation is drawn based on our analysis (**F**).

Cellular Zinc (Zn^+2^) concentrations and transport are critical for autophagy^40^. Depletion of intracellular Zn^+2^ by TPEN (*N*,*N*,*N′*,*N′*-tetrakis-(2-pyridylmethyl) ethylenediamine), but not cellular Zn^+2^ by DTPA (diethylenetriaminepentaacetic acid), ensued the induction of apoptosis in the hepatocytes^41^. We added intracellular (TPEN) and extracellular (DPTA) Zn^+2^ chelator in the *AcHE1b* OV oocyte culture, and observed significant reduction in autophagy and simultaneous induction of apoptosis in the former, while no distinct changes were recorded in the latter (Fig. 5A–E). Interestingly, zinc inhibits Bax and Bak activation, cytochrome c release and other subsequent steps of apoptosis^42^ and favours AMPK activation^43^, thus triggering ULK-Beclin-LC3 mediated autophagy pathway. Moreover, *P53* OV oocytes displayed extensive degeneration along with slight induction of *AcHE1b*, while *Beclin1* OV oocytes did not show this induction (data not shown). Notably, different oocyte stages exhibited a ubiquitous expression of *Beclin1* and *LC3a*, while *P53* was only observed in the mature oocytes (Supplementary Fig. 4). This confirms that *AcHE1b* is present in various stages of oocytes and triggers autophagy, and further protects the fish from extensive oocyte degeneration, as and when required.

## Discussion

Genome duplication and differential alternative splicing are the two very important factors in teleostean functional diversification, that affects the expression, sub-cellular distribution, and functional activities of various gene products^14, 15, 28^. Unlike *AcHE1b*, other *AcHE*s did not show any sex-biased expression, which suggests their conserved role in several other sexually unrelated areas, i.e. hatching and sperm-oocyte interactions^30, 44^. The evolutionarily primitive status of Japanese anchovy, the complex exon-intron interaction-based diversification of HEs^45^ and the alternative splicing of *AcHE1* likely emphasizes on the initiation of a neofunctionalization event. Follicular atresia and oocyte degeneration are well-known female-specific phenomena^13, 46^. Ovary-specific expression, female-biasness and steroid-responsive demethylation of *AcHE1*-intron-3, along with strong correlation with atretic oocytes, makes *AcHE1b*, a prime candidate for ovarian atresia regulation in Japanese anchovy. The ectopic OV and KD of *AcHE1b*, respectively, mimicked the two different modes of PCD, autophagy and apoptosis, and caused oocyte elimination.

Gonadal atresia, under both natural and stressful conditions, involves the sequential disintegration of oocyte germinal vesicles, cytoplasmic organelles and surrounding somatic cells, which are later removed by different independent machineries^2^. Previously, apoptosis was considered to be an active player in these physiological processes; however, recent biochemical and morphological evidence have revealed a more intricate involvement of autophagic cell death in oocyte elimination during atresia^35, 47^. The localization of transiently overexpressed *AcHE1b* in all stages of oocytes, along with the fact that ovarian autophagy is paramount to maintain the primordial oocyte pool in murine newborns^35, 47^, emphasizes the strong autophagy-*AcHE1b* relationship. This concept was further boosted by the strong correlation evidenced between *AcHE1b* and autophagy markers. Autophagy regulates apoptotic luteal cell death by controlling the Bax-to-Bcl-2 ratio and the subsequent activation of caspases^48^. Atresia induction or *AcHE*s OV in anchovy gonads elevated the expression of both apoptotic and autophagic genes. Furthermore, *AcHE1a*/*2a* OV gonads also showed a modest transcription of *AcHE1b*/*2b*, respectively, which together with the cogent association between *P53* and *AcHE1a*/*2a*/*2b* isoforms, implies a strong concentration responsive inter-relationship between the different *AcHE* isoforms. These interconnections eventually promote gonadal degeneration by either skipping or adding autophagy to the process.

The steroidogenic regulation of gonadal atresia is not an uncommon phenomenon among vertebrates^31, 49^. DHP and AI (aromatase inhibitor) mediated altered methylation of the *AcHE1* gene, and the strong progesterone-dependent upregulation of *AcHE1b* and autophagy markers, in addition to progesterone inducing autophagy by the mTOR pathway suppression^50^, confirms the involvement of steroids in gonadal atresia^31, 49^. In goat follicles, steroids regulate the Zn^+2^ concentrations, which is essential for hatching enzymes^51^ and autophagy^40^. Our work demonstrated the prevalence of several autophagic genes in *AcHE1b* expressing atretic oocytes. Since *AcHE*s are localized in the inner periphery of the chorion, and autophagic genes are specifically expressed in cell organelles, we presumed that some intermediary ion channel^9, 40^ or chemical cue (possibly responsive to different AcHE concentrations) is crucial for the induction of autophagy. Reversion of *AcHE1b* OV related autophagic effect by intracellular Zn^+2^ ion chelator, further strengthens the above hypothesis and also suggests that AcHE1b might be essential for zinc ion mediated autophagy maintenance in oocytes^41–43^. It is important to note that the addition of calcium (Ca^+2^) increased the germ cell survivability in medaka, reared at HT (unpublished data). The involvement of trace elements in autophagy^8, 9, 40^ and fewer cAMP-dependent protein kinase phosphorylation sites^37^ in *AcHE1b*, suggests that the *HE*s differentially trigger the uni/bidirectional ion flow and influence the cell elimination fate. Due to the differential steroid responsive transcription of *AcHE*, it is acceptable to assume that apoptosis/autophagy cell fate decision greatly depends on the alternatively splicing of HEs. However, the effects of several influencing factors, such as the nutritional and stress conditions of oocytes^36^, occurrence of follicular atresia and resorption^52^, whole body physiology^46^, ratios of different circulating steroids^49^ and the immune status of fish^15^, needs to be thoroughly investigated in order to clarify the consequences of stress on the reproductive success or failure of an organism.

## Materials and Methods

### Ethics statement

The studies were carried out in accordance with the Institutional Ethics Committee of Ehime University, Ehime, Japan, strictly adhering to the guidelines set for the usage of animals by this committee. All *in vivo* experiments and fish maintenance were conducted following protocols and procedures approved by the Institutional Animal Care and Use committee at Ehime University, Japan. All surgeries were performed under Tricaine-S anesthesia, and all efforts were made to minimize suffering.

### Experimental procedures

A detailed description of procedures used in this study is provided in Supplementary Information, Supplementary Materials and Methods.

### Plasmid construction, sequence analysis, qPCR, histology, *ISH* and western blotting

All plasmids for *ISH*, qPCR standard preparation, and *AcHE* OV and KD, were constructed following previously published methodology^53^ with slight modification, wherever necessary (details in Supplementary Materials and Methods).

cDNA sequences of AcHE alternatively spliced isoforms were obtained from Japanese anchovy gonadal cDNA. Publically available database information were combined with laboratory generated data to obtain the phylogenetic tree, signal peptide and domain analysis, protein secondary structure and phosphorylation analysis, and 3D structure prediction^15^.

Changes in gene expression were quantified using the CFX-96 Realtime PCR system (Biorad, USA) using previously published protocols^53^, with minor modifications. The PCR conditions included an initial denaturation at 94 °C (2 min) followed by 40 cycles at 94 °C (30 s) and 60 ^°^C (1 min). The geometric mean of *ef1α* and *βactin* were used to normalize the data.

Bouin fixed, paraffin embedded samples (at least 10 fish per group) were used for standard Hematoxylin & Eosin (HE) staining and fluorescent immunohistochemistry (FIHC), while, 4% paraformaldehyde fixed samples were used for *ISH*, Fluorescent *ISH* (*FISH*) and *FISH-*FIHC, using sense and anti-sense fluorescein or digoxigenin-labelled RNA probes (transcribed *in vitro*, using RNA labelling kit (Roche Diagnostics GmbH, Mannheim, Germany))^54, 55^.

300 µg crude protein extract was loaded and separated by SDS-PAGE, transferred onto a polyvinylidene fluoride (PVDF) membrane (Merck Millipore, German), blocked with PVDF blocking buffer (Toyobo, Japan), incubated with diluted antibody (1:10000) overnight, washed with TBST (20 mM tris-base, 150 mM NaCl, 0.05% tween-20, pH 7.5), incubated with 1:20000 dilution of secondary goat anti-rabbit/mouse HRP-conjugated Ab (Vector Laboratories) for 1h and band revelations were achieved by Pierce ECL Plus Western Blot Kit (Thermofisher Scientific, Japan), while imaging was done using ImageQuant LAS-4000 (GE Healthcare, USA).

### Experimental animals, designs, sample collection and data analysis

All the experiments were conducted using Adult Japanese anchovy maintained at 24 ± 2 °C, fed with commercially available feed (3 times a day, @ 4% body weight), if not otherwise mentioned, following Animal Care and Maintenance instructions of Ehime University Animal Use and Ethics Committee. Specifically, experimental groups were either starved, kept at high temperature (27 and 30 °C), fed with E2 (17β-estradiol, Wako, Japan, @ 10 mg kg^−1^ feed) or AI (aromatase inhibitor, Exemestine, GmBH, Germany, @ 100 mg kg^−1^ feed) mixed feed, injected with Progesterone (@100 ng/fish), DHP (@1 ng/fish), or transfected with *AcHE*, *P53*, *Beclin1* antisense or sense mRNA (10 ng mRNA/individual) premixed with *TransIT®-QR Delivery Solution* (Mirus-bio,USA). All the experiments were conducted for 30 days, except Progesterone and DHP (6 days), *AcHE* (10 days), and *P53* and *Beclin1* (1day, followed by 3 days of *in vitro* culture) transfection. Individual fish were biopsied to assess the gonadal maturity status and phenotypic sex, and acclimatised (2 weeks) prior to the commencement of actual experiment. Steroid doses and other conditions were determined using a series of pilot experiments. Ontogeny samplings were carried out at 4 (immature), 5 (maturing), 6 (mature) months after fertilization (maf), and one half of each gonad was used for either qPCR or histological analysis.

All experiments were conducted for a minimum of three times, and One-Way ANOVA, followed by Tukey’s test, or Student’s t-test, was performed to assess the statistical differences of relative mRNA expression (using SPSS version 22). In the starvation, high temperature, E2, AI experiment, each tanks were used as replicate; while progesterone, DHP, *AcHE* KD exposed individuals were used as replicate. All experimental data are shown as mean ± SEM. Differences were considered statistically significant at P < 0.05, if not otherwise mentioned. The correlations were calculated using Pearson correlation coefficient method.

### Methylation analysis

Sex-biased methylation specific loci were identified using specific primers (designed using MethPrimer (http://www.urogene.org/cgi-bin/methprimer/methprimer.cgi)) of *AcHE1* and *AcHE2* intronic sequences (provided by Prof. Kawaguchi). Ovarian, testicular and experimental gonadal genomic DNAs were isolated, bisulfate treated using EZ methylation kit (Zymo, USA), PCR amplified using gene specific primers, cloned and sequenced. At least 5 gonads/experimental group/biological replicate were individually assessed, while 20 clones per gonad were used for sequence comparison.

### Isolation and culture of female germ cells, overexpression of *AcHE*s, zinc ion estimation and autophagosome measurement

Germ cells and oocytes of five adult Japanese anchovy were enzymatically digested, gently suspended in L15 media (containing 1% FBS), chronologically sieved through 100, 40, 20, 5 μm mesh (Nytal, Switzerland), segregated using percoll (wherever necessary, to obtain early and late vitellogenic, immature and primary oocytes, and oogonia), separately cultured (100nos/well of 6 well dish) for 24 hours, using DMEM/F12 glutamax media (Gibco, USA) and 10% FBS (Gibco, USA), transfected with different *AcHE* overexpression plasmids (in triplicates) and analysed by confocal microscopy (Zeiss Axio 700), qPCR and histology. Controls were simultaneously prepared using empty vector transfected samples. Both isolation and transfection experiments were repeated for five times, and the averages of five different experiments were used for data analysis. Cells larger than 100 μm were further segregated into mid-vitellogenic, mature and hydrated fractions, using inverted microscope (Nikon, Japan), wherever necessary. Ovulated eggs were dissected out from five ovulating females during ovulation. Parallel experiments were conducted to ascertain the phenotypic degradation of cells (viable cell population) using tryphan blue stain (0.6 mg/ml) for 30 min at room temperature.

Similarly prepared and transfected (with *AcHE1b* OV plasmid) germ cells were treated (4 hpt) with either TPEN or DTPA (@1nM) Zn^+2^ ion Chelator^56^, and autophagic status were assessed by qPCR and FIHC. The cellular and medium zinc concentration was measured using Amplite™ Fluorimetric Zinc Ion Quantitation Kit (AAT bioquest), following manufacturer’s instructions.

Autophagosomes were measured using CYTO-ID Autophagy detection kit (Enzo, Japan), following manufacturer’s protocol. Briefly, freshly dissociated cell mixture from different treatment groups were fixed with using 2% formaldehyde for 20 min, perforated with Glycine, washed with PBS, blocked using 2% FBS-PBS-0.001% Triton X for 1h, incubated with Alexa 488 tagged LC3 (1:300) antibody overnight, washed repeatedly, mounted on glass slides for microscopic observation and photomicrographed, using Axio-710 confocal microscope (Zeiss, Germany) and finally analysed using Image J software.

## Electronic supplementary material

Supplementary Information
