# Supplementary material for: Hatching enzymes disrupt aberrant gonadal degeneration by the autophagy/apoptosis cell fate decision
Source: Sci Rep. 2017 Jun 9;7:3183. doi: 10.1038/s41598-017-03314-7 (PMC5466654; doi:10.1038/s41598-017-03314-7)

Hatching enzymes disrupt aberrant gonadal degeneration by the autophagy/apoptosis cell fate decision

Tapas Chakraborty[1,#,*], Sipra Mohapatra[1,#], Megumi Tobayama[1,#], Kayoko Ohta[1], Yong-Woon Ryu[1], Yukinori Kazeto[2], Kohei Ohta[1,3], Linyan Zhou[4,*], Yoshitaka Nagahama[1,5], Takahiro Matsubara[1,*]

1. South Ehime Fisheries Research Center, Ehime University, Ainan, 798-4206, Japan
2. National Fisheries Research Center, Oita, 879-2602, Japan
3. Laboratory of Marine Biology, Kyushu University, Fukouka 812-8581, Japan
4. Key Laboratory of Freshwater Fish Reproduction and Development, Southwest University, 400715, China
5. Institution for Collaborative Relations, Ehime University, Matsuyama, 790-8577, Japan

Short Title: Hatching enzymes initiate gonadal atresia

[#] These authors contributed equally to this work.

*Address all correspondence and requests for reprints to:

1. Dr. Tapas Chakraborty,
South Ehime Fisheries Research Center, Ehime University, Japan
Tel: 81-895-73-7112; Fax: 81-895-73-7113
Email: tchakraborty83@gmail.com

2. Dr. Linyan Zhou
Southwest University, China
Email: yanlinzhou916@126.com

3. Prof. Takahiro Matsubara
South Ehime Fisheries Research Center, Ehime University, Japan
Tel: 81-895-73-7112; Fax: 81-895-73-7113
E-mail: matsu@agr.ehime-u.ac.jp

Key words: Hatching enzymes, Alternative splicing, Oocyte degeneration, Autophagy, Gonad.

Supplementary Information:

1. **Supplementary Materials and Methods**
2. **Supplementary Table 1. Inter-specific percent similarity index of various AcHE proteins**
3. **Supplementary Table 2. Protein structure analysis of alternatively spliced AcHE1 and 2**
4. **Supplementary Table 3: Average gene expression and different oocyte populations in various experimental controls**
5. **Supplementary Table 4. Correlation between different *AcHEs* and occurrence of atresia.**
6. **Supplementary Table 5. *AcHE*- apoptosis-autophagy correlation.**
7. **Supplementary Figure 1. Spatio-temporal and phylogenetic profiling of *AcHE*s.**
8. **Supplementary Figure 2. Characterization of alternatively spliced AcHE1 and 2 isoforms.**
9. **Supplementary Figure 3. Progression of gonadal atresia in Japanese anchovy.**
10. **Supplementary Figure 4. Expression profiling of *P53*, *Beclin1* and *LC3a* in various oocytes/ovary.**

Supplementary Materials and Methods

Plasmid construction and mRNA transcription. pGEMT-easy plasmids carrying fragments of different genes were used for *in situ hybridization* (*ISH*) probe synthesis and qPCR standard preparation, wherever necessary. Expression plasmids were constructed using pCS2(+) vector backbone and infusion cloning kit (Clonetech, USA) containing the complete ORFs of required genes, if not specifically mentioned. *AcHE1*-5'-UTR, *AcHE1a/b* ORF-mCherry and *AcHe1a/b* 3`utr, *AcHE2*-5`utr, *AcHE2a/b* ORF-pmCherry and *AcHE2a/b* 3`utr were sequentially cloned into pCS2 vector, and named as pCS2-AcHE1a-mCherry-OE, pCS2-AcHE1b-mCherry-OE, pCS2-AcHE2a-mcherry-OE and pCS2-AcHE2a-mcherry-OE, respectively. The βactin 3` UTR were cloned into pCS2-pam-cyan vector after cyan-ORF and named PCS2-cyan-bact-3UTR. The *AcHE1a*, *1b*, *2a*, *2b, P53, Beclin1* antisense fragments were designed, incorporated to pcDNA 3.1 (+) vector, respectively, named as pcDNA3.1-AcHE1a, pcDNA3.1-AcHE1b, pcDNA3.1-AcHE2a, pcDNA3.1-AcHE2b, pcDNA3.1-P53, pcDNA3.1-Beclin1 and validated[1]. The overexpression (OV) and knockdown (KD) mRNA were transcribed with mMESSAGE mMACHINE T7 and SP6 kits (Ambion), following manufacturer's instructions. Poly A was added to the synthetic mRNA using Poly A tailing kit (Ambion).

Experimental animals, designs and statistical analysis. Adult Japanese anchovy were used in various experiments, if not otherwise mentioned. Generally, fish were maintained in flow-through water, at 24 ± 2°C, under a photoperiod comprising of 14h lightness and 10h darkness, fed (3 times a day, @ 4% of their body weight) with commercially available pelleted feed (Otohime, Japan), following Animal Care and Maintenance instructions of Ehime University Animal Use And Ethics Committee. Individual fish were biopsied to assess the gonadal maturity status and phenotypic sex, and acclimatised in experimental tanks (100 fish/tank) for 2 weeks prior to the commencement of actual experiments. Steroid doses and other conditions were determined using series of pilot experiments. 10 fish were sampled at each stage from each group. The experiments were conducted atleast thrice, using three different mature populations of fish.

Starvation and high temperature treatment: After the acclimation period, fish were sub-divided into eight different tanks (stocking density of 100 fish/sex/3 tonnes water). Fish in tanks 1 to 4 were fed with the commercial diet, while the fish in the

remaining four tanks were starved continuously for a period of 30 days. Similarly, in the high-temperature experiment, twelve tanks (stocking density of 100 fish/sex/3 tonnes water) were used. Four tanks each were maintained at 25^0C (control), 27^0C and 30^0C, respectively, for 30 days. Samplings were carried out at 5, 12, 20 and 30dat (days after treatment) in both the experiments. The individual fish samples were treated as pseudoreplicate and tanks were used as replicate. The experiments were conducted thrice with three distinct populations of mature fish. Tank averages of each category were used for statistical analysis.

Estrogen (E2) and Aromatase Inhibitor (AI) exposure: E2 (Wako, Japan) and AI (Exemestine, GmBH, Germany) were dissolved in ethanol, mixed with pelleted feed (10mg kg^{-1} E2 and 100mg kg^{-1} AI), dried overnight, fed to 100 male or female fish, respectively, for a period of 30 days and sampled periodically at 15 and 30dat. Vehicle controls, fed with ethanol soaked pelleted feed, were similarly maintained and sampled. The experiments were repeated three times and both design and analysis were similar to abovementioned starvation experiment.

Progesterone and Di hydroxyl-progesterone (DHP) injection: Progesterone and DHP were dissolved in methanol, injected (100ng progesterone/fish, 1ng DHP/fish) to 20 female fish each, and periodically sampled at 1 or 6 day after injection. Vehicle control, injected with 50µl of methanol/ fish was simultaneously sampled at each time points. The experiments were performed thrice and individuals were used as replicate.

Knockdown of different AcHEs and atretic genes: Antisense and sense mRNAs of different *AcHE*s were separately synthesized using *in vitro* RNA synthesis Kit (Ambion, USA), purified, dissolved in RDW (RNAse free distilled water), mixed with *TransIT®-QR Delivery Solution* (Mirus-bio, USA) and injected into 10 fish each (@ 10ng mRNA/ individual). Gonadal histology, occurrence of atresia, and hatching enzyme mRNA concentrations were assessed after 10 days of injection. Vehicle-controls were maintained and sampled simultaneously. Similarly synthesized and transfected *P53* and *Beclin1* gonads were dissected after 1 day and cultured *in vitro* for 3 days and sampled at daily intervals. The experiments were repeated four times and individuals were used as replicate.

Sample collection. Ontogeny samplings were carried out at 4, 5 and 6 month after fertilization (maf). At each stage of sampling, parts of each gonads was separately stored in RNAlater (Ambion, USA), or fixed using Bouin solution. After assessing the

gonadal sexuality and maturity status (by assessing the abundance of sperm and vitellogenic oocytes) by histological analysis of Bouin-fixed samples, 10 individuals of each sex were randomly selected and used for RNA isolation. The atretic gonads were collected from mature population of fish with high degree of oocyte degeneration. The embryos were grown at 24^0C, sampled at stage 39 (just before hatching) and stored in RNAlater for further analysis. For all experimental samples, parts of each experimental gonad were snap-freezed in liquid nitrogen (for methylation analysis), stored in PBS at -80^0C (for steroid analysis), stored in RNAlater (for qPCR analysis), fixed in Davidson fluid or Bouin (for histology and immunohistochemistry (IHC)), or processed using PFA (for *ISH*, fluorescent *ISH* (*FISH*), *FISH*-fluorescent IHC (FIHC)).

cDNA and protein analysis. Using available informations[2], various hatching enzyme cDNA and protein sequences were obtained from the NCBI (http://www.ncbi.nlm.nih.gov/) to create phylogenetic tree[3]. The putative amino acid sequences were translated using Expasy translation tools (http://web.expasy.org/translate). The putative signal peptides and domains were analyzed using InterProScan 5 (http://www.ebi.ac.uk/Tools/pfa/iprscan5). The secondary structural analysis, i.e. helix formation, helix-helix interactions, beta and gamma turns were performed using ProFunc (http://www.ebi.ac.uk/thornton-srv/databases/profunc). The phosphorylation characteristics were analyzed using KinsePhos 2 (http://kinasephos2.mbc.nctu.edu.tw). The *in silico* protein 3D structures were predicted using Swiss Modelling (http://swissmodel.expasy.org)[3].

Quantification of changes in gene expression by qPCR. Changes in gene expression were quantified using the CFX-96 Realtime PCR system (Biorad, USA). Total RNA was isolated from cells, embryos, gonads and other tissues using RNeasy Mini or micro kit (Qiagen, Germany). cDNA synthesis was carried out using iScript™ Advanced cDNA Synthesis Kit (Biorad, USA) from 1µg of total RNA. The first strand cDNAs were diluted to 200µl for subsequent use. Gene-specific qPCR was performed using 2µl of cDNA and SYBR green master mix (Applied Biosystem, USA), according to manufacturer's instructions. The PCR conditions included an initial denaturation at 94^0C (2 min) followed by 40 cycles at 94^0C (30s) and 60^0C (1 min). *Ef1α* and *βactin* were used as internal controls. The absolute transcript copy

number of each gene was determined with the help of appropriate standard curves and normalized with the geometric mean *ef1α* and *βactin* copy numbers in each sample. Average relative expression derived from triplicates was used for further analysis. The specificity of primer sets, throughout this range of detection, was confirmed by the observation of a single amplification product of the expected size, Tm (melting temperature) and sequences. All assays were quantified, with standard curves (mean Ct vs. log cDNA dilution) having slopes between -2.99 and -3.34, a linear correlation (R^2) between the mean Ct and the logarithm of cDNA dilution of >0.985 in each case. All test cDNAs were run in duplicates for each gene.

Histology, *ISH, FISH* and FIHC. Bouin fixed, paraffin embedded samples (at least 10 fish per group) were used for standard Hematoxylin & Eosin (HE) staining and FIHC, while, 4% paraformaldehyde fixed samples were used for *ISH, FISH* and *FISH*-FIHC (Fluorescent *ISH*). All the histological analyses were performed using 5μm sections. For *ISH*, sense and anti-sense flourescin or digoxigenin-labeled RNA probes were transcribed *in vitro*, using RNA labelling kit (Roche Diagnostics GmbH, Mannheim, Germany), from plasmid DNA containing respective genes. Sections were deparaffinized, hydrated, treated with proteinase K at 10μg ml^{-1} (Roche), and hybridized with the sense or anti-sense labelled RNA probes at 60^0C for 18-24h. The hybridization signals were detected following previously published protocols[4,5].

Methylation analysis. Methylation specific loci were identified using MethPrimer (http://www.urogene.org/cgi-bin/methprimer/methprimer.cgi), and gene specific primers were designed using partial *AcHE1* and *AcHE2* intronic sequences (kindly provided by Prof. Kawaguchi). The primers were used to amplify the required genomic fragments, separately, from both ovary and testis genome. The experimental gonadal genomic DNAs were isolated, bisulfate treated using EZ methylation kit (Zymo, USA), PCR amplified using gene specific primers, cloned into pGEMT-easy vector, and sequenced. At least 5 gonads from each experimental group were used for PCR amplification and cloning, while 20 clones from each gonad groups were used for sequence comparison.

Isolation and culture of female germ cells and overexpression of *AcHEs*. Five adult Japanese anchovy ovary were dissected, scrapped using a scalpel to separate the oocytes (including surrounding somatic cells), gently suspended in L15 media and treated with collagenase (0.2µg ml^{-1}) for 1h, gently mixed with glass pipette and sieved through 100µm mesh (Nytal, Switzerland). The filtrate was washed with L15 media (containing 2% FBS) and used for further analysis. The solution was mixed with Trypsin (0.25%), incubated in a rotator with gentle shaking and periodic mixing for 1h. The enzymatic reaction was disrupted using 1% FBS. The cell suspension was serially passed through 100, 40, 20, 5µm mesh (Nytal, Switzerland) to obtain different size fractions of oocytes. Pilot experiments findings suggested that atretic/mature/late-vitellogenic, early/mid-vitellogenic, chromatin nuclear/peri-nuclear, and primary oocyte/early oogonia were predominant in 100, 40, 20, 5µm fragments, respectively. Vitellogenic fraction was further sub-fractioned into early and mid-vitellogenic enriched population using 30-70% Percoll (GE healthcare) gradient (prepared using 1% PBS-FBS) centrifugation, whenever necessary. The primary oocyte and oogonia mix were separated using 10-30% Percoll gradient. 100 cells from each fractions were cultured into 6 well dish for 24h, using DMEM/F12 glutamax media (Gibco, USA) and 10% FBS (Gibco, USA). Each size fractions were co-transfected (1:1 ratio) with different *AcHE* overexpression mRNAs and *Cyan-βactin 3`UTR* mRNA, using TransIT mRNA kit (Mirus bio, USA) (in triplicates). The reporter gene expression was observed at 24 and 120hpt (hours post transfection), using Zeiss Axio700 confocal microscope. Cells from each well were separately collected in individual tubes and stored for RNA isolation and qPCR analysis. The oocyte isolation and the following transfection experiments were repeated for five times, and each repeat was used as a replicate. Controls were simultaneously prepared using empty vector transfected samples. Cells larger than 100µm were further segregated into late-vitellogenic, mature and hydrated fractions, using inverted microscope (Nikon, Japan), wherever necessary. Ovulated eggs were dissected out from five ovulating females during ovulation. Viability of size-fraction specific transfection experiment was pre-standardized using pCS2-GFP-Nanos 3'UTR mRNA. Parallel experiments were conducted to ascertain the phenotypic degradation of cells (viable cell population) using tryphan blue stain (0.6mg ml^{-1}) for 30 min at room temperature.

Intracellular zinc (Zn^{+2}) ion estimation. Different population of germ cells (prepared similarly and same time as *AcHE* OV experiment) were similarly transfected with *AcHE1b* OV mRNA. Zn^{+2} ion chelators, TPEN or DTPA (@1nM) were added 4h post transfection, and autophagic status of each oocyte population was assessed by qPCR and FIHC (after 3h). DMSO was used as vehicle control. The cellular and medium Zn^{+2} concentrations was measured using Amplite™ Fluorimetric Zinc Ion Quantitation Kit (AAT bioquest), following manufacturer's instructions.

Data analysis. Statistical differences in relative mRNA expression between various experimental groups were assessed by One or two-way ANOVA of normalized data, followed by Tukey's test, or Student's t-test. All statistical analyses were performed using SPSS, version 22. All experimental data are shown as mean ± SEM. Differences were considered statistically significant at $P<0.05$, if not otherwise mentioned. The correlations were calculated using Pearson correlation coefficient method.

References

1. Chakraborty, T., Zhou, L.Y., Chaudhari, A., Iguchi, T., Nagahama, Y. *Dmy* initiates masculinity by altering *Gsdf/Sox9a2/Rspo1* expression in medaka (*Oryzias latipes). Sci. Rep.* **6**, 19480 (2016).
2. Kawaguchi, M. *et al*. Different hatching strategies in embryos of two species, Pacific Herring *Clupea pallasii* and Japanese Anchovy *Engraulis japonicus*, that belong to the same order Clupeiformes, and their environmental adaptation. *J. Exp. Zool. B: Mol. Dev. Evol.* **312(2)**, 95-107 (2009).
3. Mohapatra, S. *et al.* Steroid responsive regulation of *IFNγ2* alternative splicing and its possible role in germ cell proliferation in medaka. *Mol. Cell. Endocrinol.* **400,** 61-70 (2015).
4. Chakraborty, T. *et al.* Different expression of three estrogen receptor subtype mRNAs in gonads and liver from embryos to adults of the medaka, *Oryzias latipes*. *Mol. Cell. Endocrinol.* **333(1)**, 47-54 (2011).
5. Zhou, L.Y. *et al.* Rspo1-activated signalling molecules are sufficient to induce ovarian differentiation in XY medaka (*Oryzias latipes*). *Sci. Rep.* **6**, 19543 (2016).

Supplementary Table 1. Inter-specific percent similarity index of various AcHE proteins

	AcHE1a	AcHE1b	AcHE2a	AcHE2b	AcHE2c	AcHE3	AcHE4	AcHE5
AcHE1a	100	**88.89**	49.27	53.31	67.28	42.65	47.06	47.79
AcHE1b		100	61.84	62.31	61.35	40.58	40.10	40.58
AcHE2a			100	**93.10**	**76**	29.09	31.27	33.10
AcHE2b				100	**76.37**	28.19	30.54	32.89
ACHE2c					100	41.10	41.78	44.18
AcHE3						100	37.12	43.14
AcHE4							100	41.46
AcHE5								100

Note: Bold values indicates similarity between alternative spliced forms.

Supplementary Table 2. Protein structure analysis of alternatively spliced AcHE1 and 2

	AcHE1a	AcHE1b	AcHE2a	AcHe2b	AcHE2c
**Secondary structure**					
Sheets	2	1	1	1	2
Beta-alpha-beta unit	1	1	1	1	1
Beta hairpin	2	1	1	1	2
PSI loop	1	1	1	1	1
Strands	7	5	5	6	7
Helix	7	5	4	7	7
Helix-helix interaction	4	4	1	2	3
Beta turns	22	19	9	15	21
Gamma turns	2	4	3	6	4
Disulphide bond	3	1	2	1	2
PORES	0	2	1	1	0
Tunnel	2	5	2	1	2
**Phosphorylation**					
cAMP-dependent protein kinase(PKA)	7	2	4	4	5

Supplementary Table 3: Average gene expression and different oocyte populations in various experimental controls

			Average gene expression normalized with geometric mean *ef1α* and *βactin*								Cells (µm)/1 mg gonad					
										>5	5 to 20	20to 40	40-100	>100	
Experiment	Content	N	*AcHE1a*	*AcHE1b*	*AcHE2a*	*AcHE2b*	*Beclin1*	*LC3*	*P53*	Oogni a	Primary oocyte	Immatur e	Vitello genic	Matur e	Atr etic
Starvation	C-5D	6	0.000140	0.000009	0.000521	0.002028	0.001541	0.003014	0.000002	125	378	243	150	110	11
	C-20D	6	0.000433	0.000033	0.000934	0.003188	0.005174	0.006494	0.000022	137	356	276	157	112	16
High temperature	C-5D	6	0.000121	0.000001	0.000504	0.001019	0.001767	0.001308	0.000003	122	384	237	138	115	13
	C-20D	6	0.000519	0.000043	0.000969	0.007462	0.008221	0.004872	0.000050	129	411	284	148	117	17
Progesteron e	C-1D	8	0.000194	0.000004	0.000456	0.002124	0.000216	0.000936	0.000004	134	396	248	164	107	13
	C-6D	8	0.000329	0.000051	0.000713	0.002455	0.000235	0.014819	0.000012	132	389	278	147	111	12
DHP	C-1D	8	0.000194	0.000004	0.000456	0.002124	0.000226	0.000836	0.000004	137	358	266	152	115	12
	C-6D	8	0.000329	0.000051	0.007413	0.002455	0.000235	0.013819	0.000009	133	365	288	156	118	11
17β estradiol	C-15D	6	0.000594	0.000044	0.000756	0.003212	0.003174	0.004494	0.000012	128	384	256	149	127	14
	C-30D	6	0.000565	0.000055	0.001350	0.004395	0.005342	0.006786	0.000034	139	376	311	160	137	18
Aromatase Inhibitor	C-15D	6	0.000194	0.000044	0.000456	0.003121	0.003917	0.004565	0.000011	135	423	264	138	128	15
	C-30D	6	0.000329	0.000051	0.001325	0.004549	0.005174	0.006456	0.000045	147	412	306	163	139	20

Note: Immature oocytes include both peri-nuclear and chromatin nuclear oocytes.

Supplementary Table 4. Correlation between different *AcHEs* and occurrence of atresia.

	Stage1	Stage2	Stage3	Stage4	Stage5	Total
AcHE1a	-0.03	0.03	0.05	0.04	0.10	0.01
AcHE1b	-0.52	0.15	0.38	0.25	0.51	-0.31
AcHE2a	-0.04	-0.33	-0.04	-0.10	0.11	-0.05
AcHE2b	0.33	-0.04	-0.03	0.08	0.22	0.43

Note: Correlations are calculated using data from steroids, starvation and HT treatments.

Supplementary Table 5. *AcHE*- apoptosis-autophagy correlation.

	AcHE1a	*AcHE1b*	*AcHE2a*	*AcHE2b*	*Beclin1*	*LC3a*	*P53*
AcHE1a	1.00	-0.23	0.61	0.37	-0.04	-0.09	**0.55**
AcHe1b		1.00	-0.07	-0.25	**0.44**	**0.30**	-0.22
AcHE2a			1.00	0.48	-0.15	-0.09	**0.53**
AcHE2b				1.00	-0.13	-0.19	**0.62**
Beclin1					1.00	0.33	-0.13
LC3a						1.00	0.05
P53							1.00

Note: Correlations are calculated using data from steroids, starvation and HT treatments.

Supplementary Figures:

Supplementary Figure 1. Spatio-temporal and phylogenetic profiling of *AcHE*s. FIHC staining, using eel hatching enzyme antibody, depicted ubiquitous fluorescence in both testis (A) and ovary (B). Different stages of oocyte (Early- EO; Atretic- AO) and follicular cell (FC) candidates are marked. QPCR analysis of *AcHE1* and *2* demonstrated maximum mRNA expression in adults, while predominant transcription of *AcHE3*, *4* and *5* were seen in the embryos (C). QPCR analysis using isolated mature, hydrated and ovulated oocyte RNA (10 numbers/group) revealed significant upregulation of *AcHE3*, *4*, *5*, but not *AcHE1* and *2* (D). Phylogenetic tree emphasized the evolutionary closeness between different AcHE isoforms (E). Note: Data are presented as mean ± SEM of 10 individuals/pooled samples, and different letters (a, b, etc.) denotes significant differences at P<0.05.

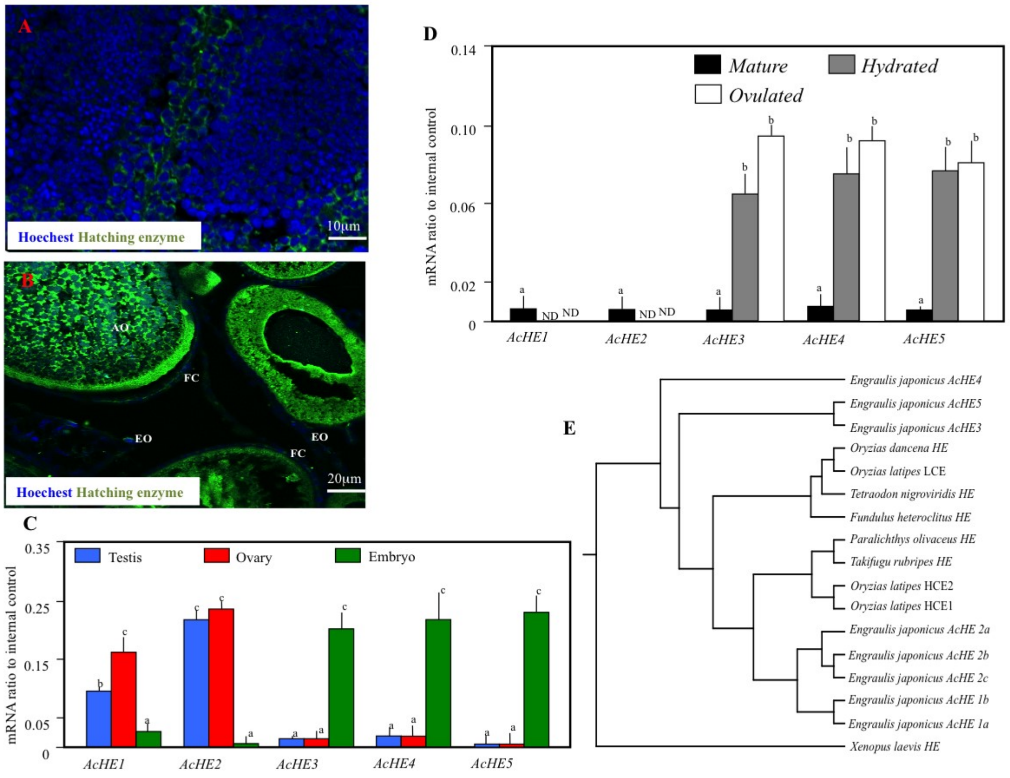

Supplementary Figure 2. Characterization of alternatively spliced AcHE1 and 2 isoforms. Quantitative tissue distribution depicted gonadal abundance of all tested isoforms, while only *AcHE1b* showed female specific expression profile (A), N=7 individual per sex. *ISH* analysis uncovered the cellular localization of *AcHE1a* (B), *AcHE1b* (C), and *AcHE2a* (D) in the inner periphery of different atretic oocytes. Gonadal ontogeny by qPCR demonstrated the variable expression profiles of AcHE spliced variants (E), N=8 per sex. Despite occurrence of variable methylation in *AcHE1*-intron-3 and *AcHE2*-intron-5 (Fig. 2), the preceding introns failed to reveal any difference among the sexes (F), N=15 individual per sex. Note: Data represents mean ± SEM, and different letters (a, b, etc.) or asterics ('*') denotes significant differences at $P<0.05$; schematic diagram represents the generalized methylation profiles of each intron.

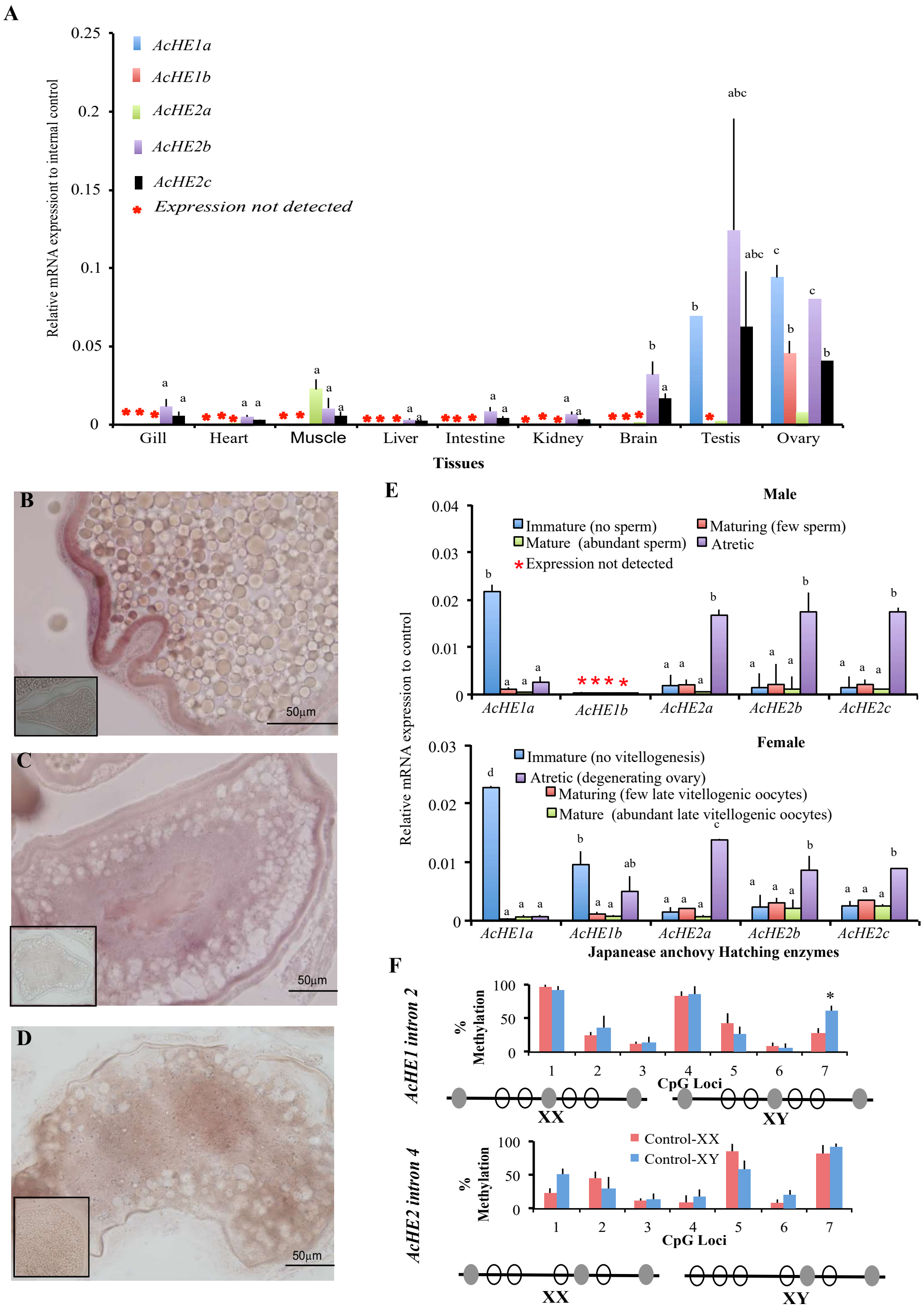
A
0.25
0.2
0.15
0.1
0.05
0
Relative mRNA expressiont to internal control
AcHE1a
AcHE1b
AcHE2a
AcHE2b
AcHE2c
Expression not detected
Gill
Heart
Muscle
Liver
Intestine
Kidney
Brain
Testis
Ovary
Tissues
abc
b
c
B
50µm
C
50µm
D
50µm
E
Male
0.04
0.03
0.02
0.01
0
Immature (no sperm)
Maturing (few sperm)
Mature (abundant sperm)
Atretic
Expression not detected
Relative mRNA expression to control
AcHE1a
AcHE1b
AcHE2a
AcHE2b
AcHE2c
Female
Immature (no vitellogenesis)
Atretic (degenerating ovary)
Maturing (few late vitellogenic oocytes)
Mature (abundant late vitellogenic oocytes)
Japanease anchovy Hatching enzymes
F
AcHE1 intron 2
% Methylation
100
50
0
1
2
3
4
5
6
7
CpG Loci
XX
XY
AcHE2 intron 4
Control-XX
Control-XY
CpG Loci
XX
XY

Supplementary Figure 3. Progression of gonadal atresia in Japanese anchovy. Pre-atretic (A), Stage 1 (B), Stage 2 (C), Stage 3 (D), Stage 4 (E), and Stage 5 (F) atretic oocytes showed different degree of granulosa cell proliferation (representative locations are denoted by ‘#’), chorion breakage (marked with ‘*’), and oocyte degradation.

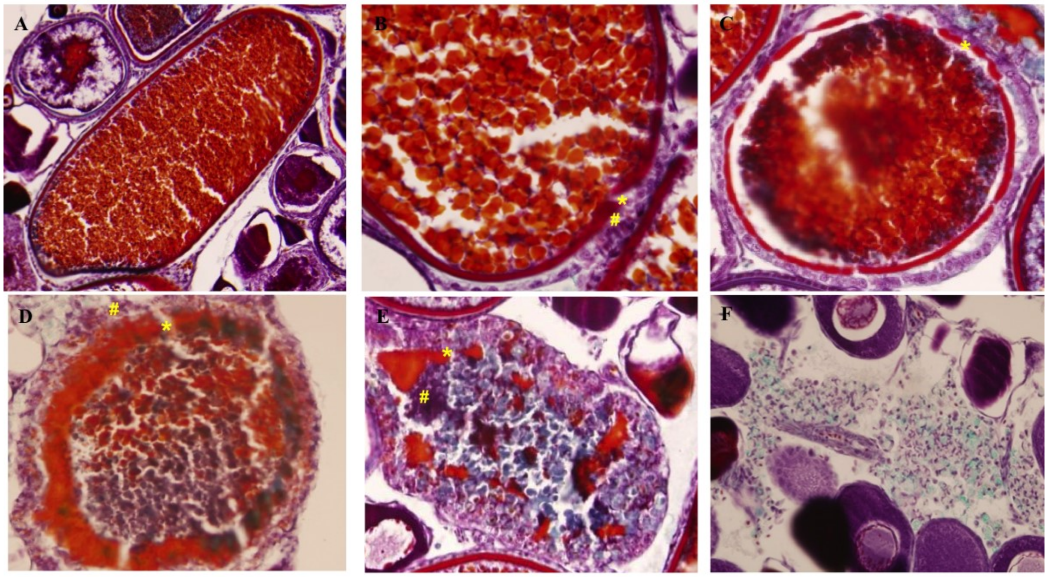

Supplementary Figure 4. **Expression profiling of *P53*, *Beclin1* and *LC3a* in various oocytes/ovary.** Differential expression characteristics in various stages of oocytes (A), steroid induced atretic gonad (B), and *AcHE1a* (C), *AcHE1b* (D), *AcHE2a* (E) and *AcHE2b* (F) over-expressed oocytes was measured by qPCR. Data are presented as mean ± SEM, and the letters (a, b, etc.) indicate that these groups differ significantly from each other at P<0.05. Note: In graphs, '*' represents non-detectable expression, VTG (vitellogenesis).

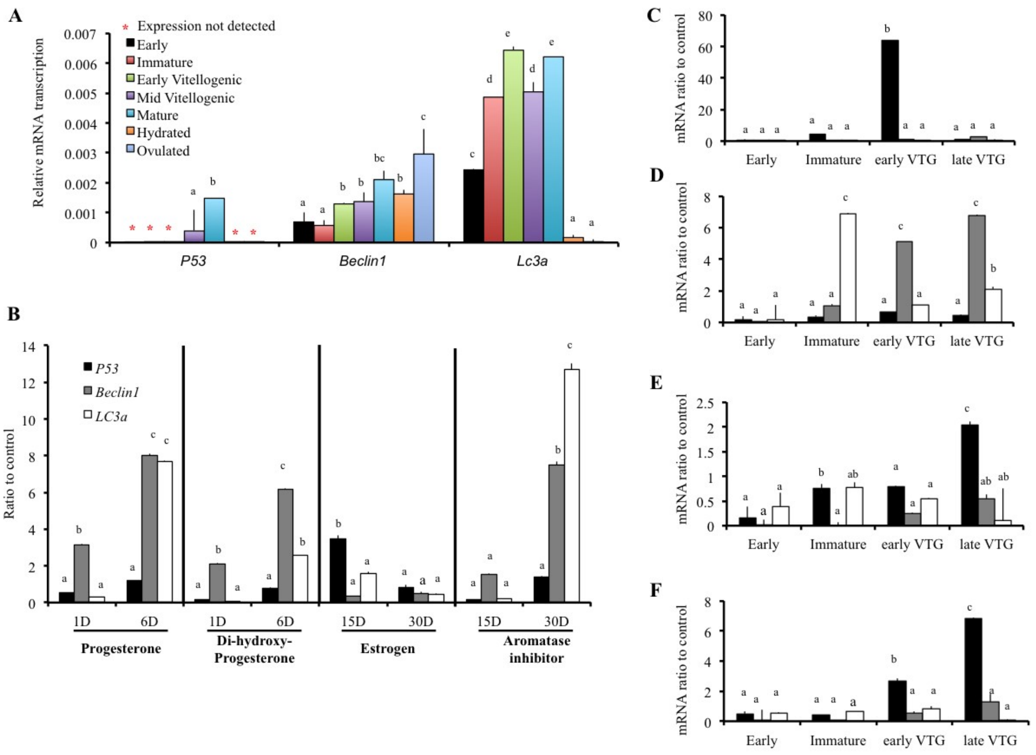

Supplement: Supplementary file 1 — Supplementary Information [file 41598_2017_3314_MOESM1_ESM.pdf]
